# Decreased expression of GEM in osteoarthritis cartilage regulates chondrogenic differentiation via Wnt/β-catenin signaling

**DOI:** 10.1186/s13018-023-04236-z

**Published:** 2023-10-04

**Authors:** Lu Gan, Zhonghao Deng, Yiran Wei, Hongfang Li, Liang Zhao

**Affiliations:** 1 Department of Orthopaedic Surgery, Nanfang Hospital, Southern Medical University, Guangzhou, 510515 Guangdong China; 2 Guangdong Provincial Key Laboratory of Construction and Detection in Tissue Engineering, Southern Medical University, Guangzhou, 510515 Guangdong China; 3 Yijiandian Clinic, Beijing, 100033 China

**Keywords:** GEM, Wnt/β-catenin signaling, Chondrogenesis, Osteoarthritis

## Abstract

**Background:**

GEM (GTP-binding protein overexpressed in skeletal muscle) is one of the atypical small GTPase subfamily members recently identified as a regulator of cell differentiation. Abnormal chondrogenesis coupled with an imbalance in the turnover of cartilaginous matrix formation is highly relevant to the onset and progression of osteoarthritis (OA). However, how GEM regulates chondrogenic differentiation remains unexplored.

**Methods:**

Cartilage tissues were obtained from OA patients and graded according to the ORASI and ICRS grading systems. The expression alteration of GEM was detected in the Grade 4 cartilage compared to Grade 0 and verified in OA mimic culture systems. Next, to investigate the specific function of *GEM* during these processes, we generated a *Gem* knockdown (*Gem*-Kd) system by transfecting siRNA targeting *Gem* into ATDC5 cells. *Acan*, *Col2a1*, *Sox9*, and Wnt target genes of *Gem*-Kd ATDC5 cells were detected during induction. The transcriptomic sequencing analysis was performed to investigate the mechanism of *GEM* regulation. Wnt signaling pathways were verified by real-time PCR and immunoblot analysis. Finally, a rescue model generated by treating *Gem*-KD ATDC5 cells with a Wnt signaling agonist was established to validate the mechanism identified by RNA sequencing analysis.

**Results:**

A decreased expression of *GEM* in OA patients’ cartilage tissues and OA mimic chondrocytes was observed. While during chondrogenesis differentiation and cartilage matrix formation, the expression of *GEM* was increased. *Gem* silencing suppressed chondrogenic differentiation and the expressions of *Acan*, *Col2a1*, and *Sox9*. RNA sequencing analysis revealed that Wnt signaling was downregulated in *Gem*-Kd cells. Decreased expression of Wnt signaling associated genes and the total β-CATENIN in the nucleus and cytoplasm were observed. The exogenous Wnt activation exhibited reversed effect on *Gem* loss-of-function cells.

**Conclusion:**

These findings collectively validated that GEM functions as a novel regulator mediating chondrogenic differentiation and cartilage matrix formation through Wnt/β-catenin signaling.

**Supplementary Information:**

The online version contains supplementary material available at 10.1186/s13018-023-04236-z.

## Introduction

Osteoarthritis (OA) affects over 300 million population worldwide [1]_,_ which causes physical disability, decreased quality of life, and increased mortality among the elderly [2]. Reduced articular cartilage and dysfunction of cartilage reformation are key changes in the pathogenesis and progression of OA [3]. Chondrogenic differentiation and the formation of the extracellular matrix, contributing to the formation of a healthy microenvironment in articular cartilage, are suppressed in OA [4–6]. However, the mechanism underlying the dysfunction of cartilage regeneration in OA has not been fully explained [1, 7]. In a previous investigation, among the differentially expressed genes of knee articular chondrocytes between OA and normal donors, *GEM* was found to be expressed significantly less in the chondrocytes of OA donors.

*GEM*, also known as GTP-binding protein overexpressed in skeletal muscle, belonged to the RGK subfamily of small GTP-binding proteins [8]. The RGK subfamily of small GTP-binding proteins consist of four members, *REM*, *REM2*, *RAD*, and *GEM* [9], that function as powerful inhibitors of voltage-dependent calcium channels (VDCCs) and active regulators of actin cytoskeletal dynamics [10, 11]. As integral components of signal transduction cascades, the RGK family contributes to almost every aspect of cellular physiology [11]. Furthermore, it was observed to inhibit the expression of connective tissue growth factor in cardiomyocytes by binding to CCAA T-enhancer binding protein-δ (C/EBP-δ) to regulate extracellular matrix (ECM) production [12]. ECM-rich cartilage tissue is produced solely by chondrocytes, which produce large amounts of ECM molecules during development [13]. *GEM* contains a G3 GTP-binding motif, extensive amino- and carboxyl-terminal extensions outside the Ras-related domains, and a motif responsible for membrane association [14]. As a result of its conservation, the carboxyl terminus plays an important role in subcellular distribution and protein–protein interaction to control both Ca^2+^ channel activity and cytoskeletal reorganization [11]. Previous studies have reported that multiple biological functions are carried out by GEM protein, including forming the plasma membrane’s inner face, receptor-mediated signaling, the pathogenesis of glaucoma [15], and synapse development [16]. Hence, *GEM* may be involved in the development of chondrocyte. Recently, RGK proteins have also been discovered to play a role in regulating cell differentiation [17]. However, there are few studies regarding the effects of *GEM* on chondrogenic differentiation.

The present study showed a lower level of *GEM* in the articular cartilage of OA patients. Consistently, cells treated with tert-butyl hydroperoxide (TBHP) to model OA also exhibited decreased expression of *GEM*. We also found that the *GEM* expression level was elevated during chondrogenic differentiation and cartilage matrix formation. Thus, knocking down the expression of *Gem* results in suppression of chondrogenic differentiation. Moreover, bulk RNA sequencing analysis revealed that the mechanism mediating chondrogenic differentiation by *Gem* was associated with Wnt/β-catenin signaling. *Wnt* signaling activation reverses the suppression of chondrogenic differentiation in *Gem* knockdown cells. According to these findings, an innovative cartilage matrix formation regulator*,Gem,* controls the chondrogenic differentiation through Wnt/β-catenin mechanisms to maintain healthy articular cartilage.

## Methods

### Human tissue preparation

Human tissues of femoral and tibial articular cartilage were obtained from nine OA patients (6 females [age: 61.3 ± 3.0 years] and 3 males [age: 63.7 ± 1.2 years]) undergoing total knee arthroplasty at NanFang Hospital. Unilateral articular cartilage was artificially divided into six regions and graded according to the OARSI and ICRS grading systems (Additional file 1: Fig. S1A, B). The cartilage of Grade 0 and Grade 4 was isolated for the following experiments. This study was reviewed by the Ethics Committee of Southern Medical University NanFang Hospital (ref. NFEC-2020–166). Donors gave their informed consent to have their anonymized tissues used for scientific research purposes.

### Paraffin sections

All cartilage samples were fixed in 4% paraformaldehyde solution at 4 °C for 24 h. These fixed tissues were subjected to decalcification in 0.5 M EDTA for 16 weeks before dehydration, paraffin embedding, and serial sectioning. Paraffin sections with a thickness of 5 μm were acquired for subsequent staining.

### Safranin O staining and immunofluorescent staining

Safranin O staining was performed using a commercial staining kit (Solarbio) according to the manufacturer’s instructions. The OARSI grading system was used to evaluate the sections [18]. For immunofluorescence (IF) staining, prepared sections were incubated with anti-MMP13 (1:100 dilution, SantaCruz Biotechnology) and anti-GEM(1:100 dilution, SantaCruz Biotechnology) primary antibodies. Alexa Fluor 488-conjugated antibodies (Invitrogen) were used as secondary antibodies. All samples were counterstained with 4′,6-diamidino-2-phenylindole (DAPI, Abcam). A BX63 confocal microscope (Olympus) was used to perform imaging. Images were quantified by ImageJ software (NIH).

### Cell lineage culture

C28/I2(Cat#: bio-133595) and ATDC5(Cat#: bio-105955) cell lineages were purchased from ATCC company. Cells were cultured in monolayer in growth media (DMEM/F12 (Gibco) with 10% FBS (Gibco) and 1% penicillin and streptomycin (P/S, Gibco)) in a humidified atmosphere of 5% CO_2_ at 37 °C for expansion. Cells meet a confluence of 80% to 90% were trypsinized for passage culture or subsequential experiments.

### Mouse primary chondrocyte isolation and culture

On postnatal day 5 of C57BL/6 mice, articular chondrocytes were isolated from cartilage tissue for primary cultures. The knee joint area connecting the femur and tibia was chopped and digested with 1%pronase (10165921001, Roche) for 1 h and 1%collagenase (C6885-5G, Sigma-Aldrich) in DMEM for 3 h at 37 °C. The digested solution was filtered through a 40-mm cell strainer, and only cells that passed through were collected. The cells were washed with DMEM with 10% FBS and 1% penicillin–streptomycin. Next, the chondrocytes were suspended and seeded into 25 cm^2^ flasks with DMEM/F12 (10% FBS, 1% penicillin/streptomycin) at 37° C in an atmosphere containing 5% CO_2_. These cells were considered passage 0 (P0).

### Micromass culture and induction

For micromass culture, ATDC5 or C28/I2 cells were cultured in DMEM/F12 (Gibco) with 10% FBS (Gibco) and 1% penicillin and streptomycin (P/S, Gibco). Cells were maintained in a humidified atmosphere of 5% CO_2_ at 37 °C. Cultured ATDC5 chondrocytes were harvested using 0.25% trypsin–EDTA (Gibco). One droplet (20 μl) containing ATDC5 cell suspension (1 × 10^7^ cells/ml) was carefully placed in the center of each well of a 24-well plate (ABC biochemistry). After cell attachment for 3 h, 500 μl of aMEM containing 100 nM dexamethasone (Sigma-Aldrich), 100 μM ascorbate-2-phosphate (Sigma-Aldrich), and human transforming growth factor–b1 (10 μg/ml; Gibco) was added. At 3, 7, 14, and 21 days of differentiation, total RNA was extracted or micromass samples were fixed using 4% paraformaldehyde (PFA).

### TBHP treatment

To mimic OA pathogenic alteration in vitro, we incubated C28/I2 cells, ATDC5 cells, and mouse primary chondrocytes with TBHP (Macklin). Chondrocytes were seeded at a density of 3 × 10^4^ cells/well into 24-well plates, and grown to a confluence of 90%.Then 0,200,400 uM TBHP was added into the test wells and DMEM medium added into the control wells, each with three replication wells, followed by incubation for 24 h. At 4 h and 24 h of treatment, total RNA was extracted and toluidine blue staining was performed.

### CCK‑8 assay

Chondrocytes (5 × 10^3^ cells/well) were seeded into 96-well plates and cultured at 37 °C for 24 h. After the chondrocytes were stimulated with TBHP (Macklin) for 4 h and 24 h, the medium was removed. Then, 10 µL of CCK-8 (Dojindo) solution was added to each well and incubated for another 2 h at 37 °C. Finally, absorbance was measured at 450 nm using a microplate reader.

### Alcian blue staining

ATDC5 micromasses were washed with PBS and fixed with ice-cold methanol for 30 min at 4 °C. Micromasses were then incubated in Alcian Blue (AB) (0.1% AB 8GX, Sigma) for a day. Stained micromasses were washed with distilled water three times and air-dried. Micromasses were imaged with an IX73 microscope (Olympus).

### Toluidine blue staining

C28/I2 micromasses were washed with PBS and fixed using 4% paraformaldehyde (PFA) for 15 min at room temperature. Prepare the toluidine blue solution comprising 2 g of toluidine blue crystals (Sigma) and 100 ml distilled water. Micromasses were then incubated in toluidine blue for 30 min at room temperature. Stained micromasses were washed with distilled water three times and air-dried. Micromasses were imaged with an IX73 microscope (Olympus).

### In vitro gene silencing

Small interfering RNAs targeting Gem (si-Gem, siBDM1999A) were obtained from RiboBio. One droplet (20 μl) containing ATDC5 cell suspension (1 × 10^7^ cells/ml) was carefully placed in the center of each well of a 24-well plate (ABC biochemistry). Cell transfection was performed using Lipofectamine™ 3000 (Invitrogen) according to the manufacturer’s instructions. Knock-down efficiency was assessed by quantitative RT-PCR. At 3, 7, 14 and 21 days of differentiation, total RNA was extracted or micromass samples were fixed using 4% paraformaldehyde (PFA).

### Wnt/β-catenin agonist treatment

The Control and *Gem*^k/k^ ATDC5 micromasses were gently seeded in the center of each well of 24-well plates (ABC biochemistry) and treated with 0.01 μM exogenous WAY-262611(a Wnt/β-catenin agonist, Selleckchem). DMSO treatment was used as control. ATDC5 cells were therefore divided into 4 groups, including control (negative siRNA treated) + DMSO group, control + WAY group, *Gem*^k/k^ + DMSO group, and *Gem*^k/k^ + WAY group. Total RNA was extracted 7 and 14 days after chondrogenesis induction.

### RNA-seq and bioinformatics analysis

At day 21 of chondrogenesis induction, total RNA from negative siRNA treated (Control) and si-Gem treated (Gem-Kd) ATDC5 cells was extracted by SteadyPure Quick RNA Extraction Kit(Accurate Biology) following the manufacturer’s instructions. Total RNA was sent to a qualified facility for library construction. High-throughput sequencing was performed using the Illumina Novaseq 6000 (USA). The RNA-seq reads were aligned to the mouse genome (GRCm39, http://asia.ensembl.org/Mus_musculus/Info/Index) using HISAT2. StringTie was subsequently used to count reads in features [19]. Genes with low counts (< 10 in all conditions) were filtered from downstream analyses using DESeq2, in R. Count data after regularized logarithm (rlog) transformation was used for PCA analysis and plotting [20]. Benjamini–Hochberg false discovery rate (FDR) procedure was used to correct for multiple testing. Genes with FDR < 0.05 and fold change > 2 were identified as significantly differentially expressed genes (DEG) between conditions using the DESeq2 analysis of two RNA-seq biological replicates. Volcano plot was generated by ggplot2 package in R. Heatmaps were generated by the pheatmap package in R. Pathway analysis was performed using KEGG by clusterProfiler [21, 22], input with the genes that were more highly expressed in Control group than Gem-Kd ATDC5 cells (> twofold, FDR < 0.05). Enriched pathways were ranked based on the adjust *p*-value calculated by the software. Gene set enrichment analysis (GSEA) was performed using GSEA software (version 4.3.2) following the manufacturer’s instructions, input with normalized count matrix generated by the BiocGenerics package in R [23–25].

### Real-time PCR

Total RNA was isolated from articular chondrocytes or micromasses using TRIzol Reagent (Ambion). Complementary DNA was synthesized using the RevertAid First Strand cDNA synthesis kit (Thermo Fisher Scientific). Quantitative polymerase chain reaction (PCR) analyses were carried out as described using Maxima SYBRgreen qPCR master mix system (Thermo Fisher Scientific). All primers used are shown in Additional file 1: Table S1. Relative gene expression was calculated using the 2(−∆∆Ct) method. All experiments were performed in triplicate.

### Immunoblot analysis

The nuclear proteins were isolated using a Nuclear Extraction kit (Solarbio). Proteins (10 μg) were separated with 8–12% sodium dodecyl sulfate–polyacrylamide gel electrophoresis (SDS-PAGE) and then transferred onto polyvinylidene difluoride membranes (Beyotime). Membranes were incubated overnight at 4 °C with primary antibodies specific to β-CATENIN, COL2A1, GAPDH, and LAMINB (1:1000 dilution, Beyotime). After washing with TBST (Tris-buffered saline with Tween 20) thrice, the blots were incubated with corresponding secondary antibodies with 5% BSA for 1 h at room temperature. Blotting signals were detected using Near-infrared Imaging System (Odyssey).

### Statistical analysis

Unless otherwise specified, group comparisons were performed using t test when two groups were compared, and one-way ANOVA when three or more groups were compared. Statistical analyses were performed by using the GraphPad Prism version8.2.1 or R. All bar graphs represent mean ± SEM. All *p*-value were denoted as * for *p* < 0.05, ** for *p* < 0.01, *** for *p* < 0.001, **** for *p* < 0.0001. The value of *p* < 0.05 was deemed significant.

## Results

### Decreased expression of GEM in OA cartilage tissues and the OA mimic cell model

**Fig. 1:**
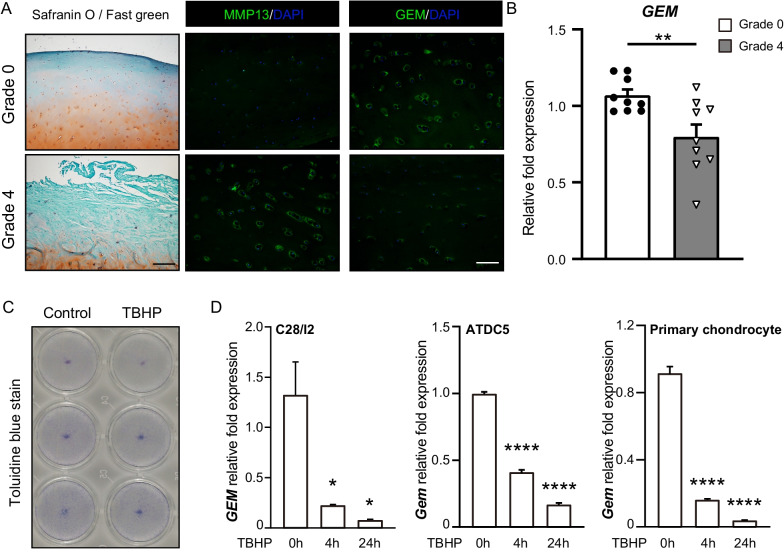
Decreased expression of GEM in OA cartilage tissues and OA mimic cell model. **A** Representative images of safranin O staining and immunofluorescence staining of GEM and MMP13 in Grade 4 and Grade 0 human knee articular cartilage tissues (*n* = 9). **B** The expression levels of GEM were quantified by real-time PCR in Grade 4 and Grade 0 cartilage tissues. **C** Toluidine blue staining indicating extracellular matrix degradation of C28/I2 cells treated with TBHP. **D** The expressions of GEM of C28/I2 cells, ATDC5 cells, and primary mouse chondrocytes at 0, 4, and 24 h after treated with TBHP. All experiments were repeated for three times. All data are expressed as mean ± SEM. *p < 0.05, **p < 0.01, ****p < 0.001, ****p < 0.0001

To verify the expression of GEM in OA cartilage, we obtained knee articular cartilage tissues from nine OA patients undergoing TKA surgery. Unilateral articular cartilage was artificially divided into six regions and graded according to the OARSI and ICRS grading systems (Additional file 1: Figure S1A, B). The cartilage tissue of Grade 4 (OA) was used to compared with that of Grade 0 (normal). The OA group showed reduced staining of Safranin O and severe cartilage destruction in contrast to the normal group (Fig. 1A). Consistently, the Mankin score of the OA group was significantly higher (Additional file 1: Figure S1C). Immunofluorescence staining results showed that in contrast to the normal group, MMP13 levels were significantly higher and GEM levels were lower in the OA group (Fig. 1A, Additional file 1: Figure S1D, E). The expression of *GEM* was also decreased in OA cartilage (Fig. 1B). The expressions of other members of RGK family such as *RRAD* and *REM* exhibited no significant difference (Additional file 1: Figure S1F). To mimic OA pathogenic alteration in vitro, we incubated C28/I2 cells, ATDC5 cells and mouse primary chondrocytes with TBHP for 24 h. The CCK-8 assay illustrated that TBHP was cytotoxic to chondrocytes, with a time-dependent decline in cell viability (Additional file 1: Figure S1G). In addition, toluidine blue staining revealed that the extracellular matrix was decreased in TBHP-treated C28/I2 cells (Fig. 1C). The expressions of *GEM* were significantly decreased overtime in TBHP-treated C28/I2 cells, ATDC5 cells, and mouse primary chondrocytes (Fig. 1D). Altogether, these results showed significantly lower expression of GEM in OA chondrocytes.

### Elevated expression of *GEM* during chondrogenic differentiation and cartilage matrix formation

**Fig. 2:**
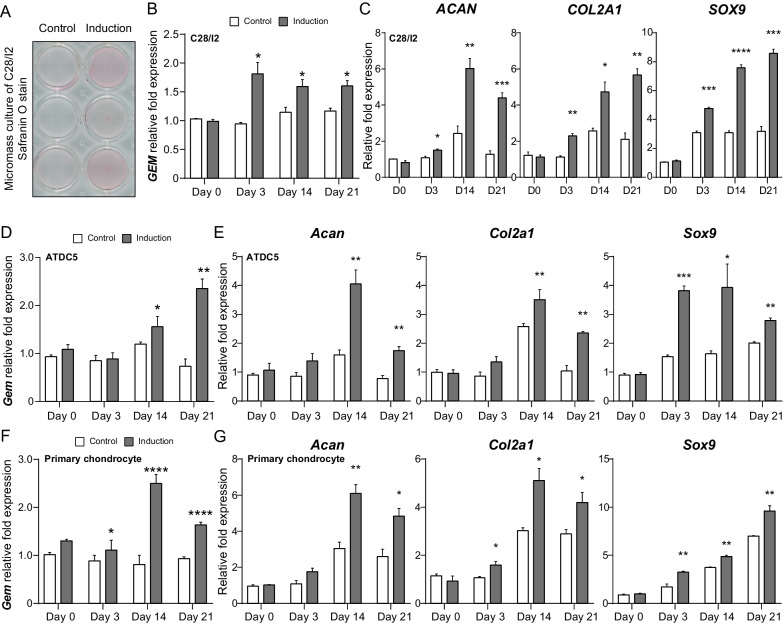
Elevated expression of GEM during chondrogenic differentiation and cartilage matrix formation. **A** Safranin O staining of C28/I2 at micromass 14 days after induction. Red color represents as positive staining. **B**, **D**, **F** The mRNA expressions of GEM among C28/I2 cells (**B**), ATDC5 cells (**D**), and mouse primary chondrocytes (**F**) 3, 14, and 21 days after induction. **C**, **E**, **G** The mRNA expressions of chondrogenic markers including ACAN, COL2A1, and SOX9 among C28/I2 cells (**C**), ATDC5 cells (**E**) and mouse primary chondrocytes (**G**) 3, 14, and 21 days after induction. All experiments were repeated for three times. All data are expressed as mean ± SEM. *p < 0.05, **p < 0.01, ****p < 0.001, ****p < 0.0001

To reveal the physiological function of *GEM* on cartilage, we established a chondrogenic differentiation model using ATDC5 cell lineages and a cartilage matrix formation model using C28/I2 cell lineages and mouse primary chondrocytes at micromass culture. By staining with Safranin O and detecting the expressions of chondrogenic markers such as ACAN, COL2A1, and SOX9, we verified the chondrogenic differentiation (Fig. 2A, C, E, G). The expressions of *GEM* were increased on Days 3, 14, and 21 (Fig. 2B, D, F) suggesting an elevated expression of *GEM* during chondrogenic differentiation. Together with the decreased GEM expressions found in the OA chondrocytes, these findings uncovered that GEM was strongly associating with cartilage pathological changes in OA.

### *Gem* silencing suppresses chondrogenesis and cartilage matrix formation

**Fig. 3:**
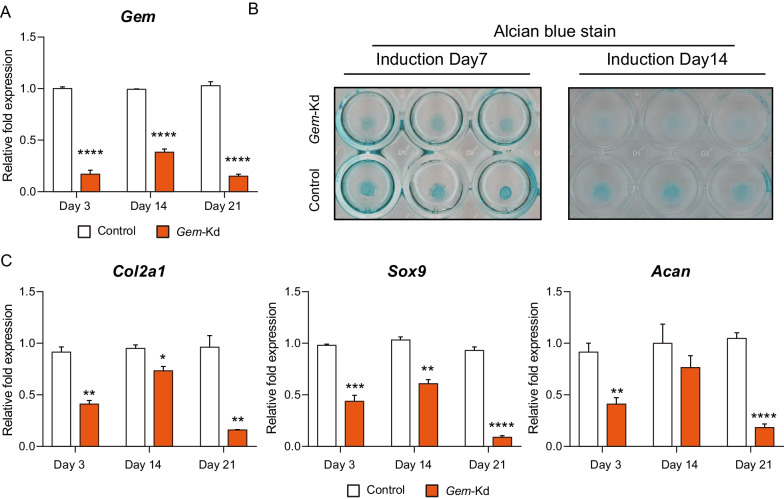
GEM silencing suppressed chondrogenesis and cartilage matrix formation. **A** The mRNA expression of Gem in control ATDC5 cells transfected with negative control siRNA (Control) and siRNA targeting Gem (Gem-Kd) 3, 14, and 21 days after chondrogenic induction. **B** Alcian Blue staining of Control and Gem-Kd cells 7 and 14 days after chondrogenic induction. **C** The mRNA expressions of chondrogenic markers including Acan, Col2a1, and Sox9 among Control and Gem-Kd cells 3, 14, and 21 days after chondrogenic induction measured by real-time PCR. All experiments were repeated for three times. All data are expressed as mean ± SEM. *p < 0.05, **p < 0.01, ***p < 0.001, ****p < 0.001

*Gem* loss-of-function model was established by transfecting siRNA targeting *Gem* into ATDC5 cells (*Gem*-Kd or *Gem*^k/k^). The silencing RNA with highest knockdown efficiency was screened priorly (Additional file 1: Figure S2A). Cells transfected with negative control siRNA were considered as control in the subsequential *Gem* silencing experiments. During chondrogenic differentiation, the expressions of Gem were decreased over time (Fig. 3A). Consistently, Gem-Kd micromasses were stained with less alcian blue (Fig. 3B, Additional file 1: S2B). The expressions of *Col2a1*, *Sox9*, and *Acan* exhibited similar trend as that of *Gem* in *Gem*-Kd cells, revealing significant decrease (Fig. 3C). Collectively, these results indicated that *Gem* silencing resulted in suppression of chondrogenesis and cartilage matrix formation in ATDC5 cells.

### Association between GEM knockdown and Wnt signaling during chondrogenic differentiation

**Fig. 4:**
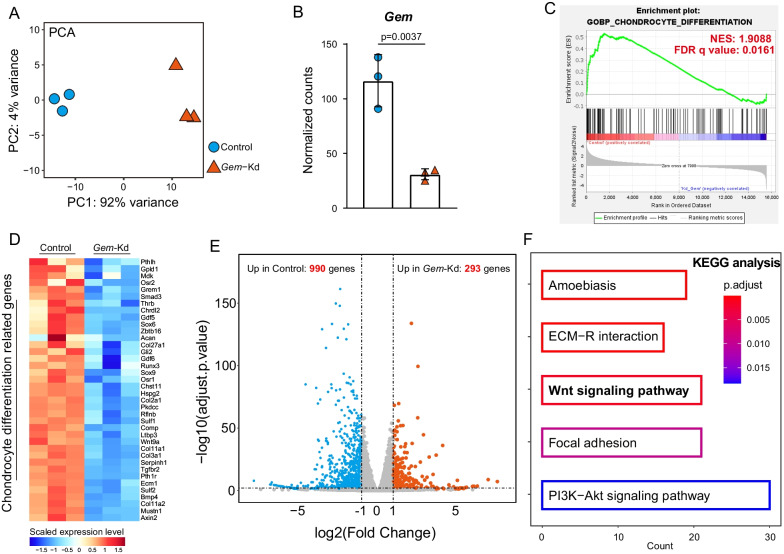
Association between GEM knockdown and Wnt signaling during chondrogenic differentiation. **A** Principal component analysis (PCA) of the transcriptomes of Control and Gem-Kd samples (n = 3 per group). **B** Normalized counts of Gem in Gem-Kd samples compared to Control samples. *p* value = 0.0037. **C** Gene set enrichment analysis (GSEA) of differentially expressed genes (DEGs) from Control and Gem-Kd cells ranked by NES scores. **D** Scaled expression levels of selected chondrocyte differentiation-related genes were showed by heatmap. **E** Volcano plot of RNA-seq analysis of DEGs using the mRNAs isolated from the Control and Gem-Kd cells. Blue dots show genes more highly expressed in Control cells than Gem-Kd cells with significant (FDR < 0.05) and greater than twofold changes. Red dots show genes more highly expressed in Gem-Kd cells than Control cells with significant (FDR < 0.05) and greater than twofold changes. **F** KEGG enrichment analysis of DEGs upregulated Control cells

To investigate the mechanisms underlying the regulatory role of the *Gem* during chondrogenic differentiation and cartilage matrix formation, we performed transcriptome RNA sequencing analysis using control (negative siRNA treated) and *Gem*-Kd ATDC5 cells after 21 days of chondrogenic induction. The principal component analysis (PCA) showed that transcriptomic profiles of control and *Gem*-Kd samples were well separated (Fig. 4A). Normalized counts of the Gem gene were significantly lower in the *Gem*-Kd group (Fig. 4B). According to gene set enrichment analysis (GSEA), genes correlated with chondrocyte differentiation were downregulated in *Gem*-Kd cells (Fig. 4C). The heatmap indicated that the scaled expression levels of selected chondrocyte differentiation-related genes were lower in the 3 replicate groups of *Gem*-Kd cells (Fig. 4D). Thus, the RNA sequencing data exhibited consistent results with the real-time PCR and safranin O staining described previously. Genes with a fold change > 2 and an FDR < 0.05 were considered differentially expressed genes (DEGs). A total of 990 genes were upregulated in Control cells, and 293 were upregulated in *Gem*-Kd cells (Fig. 4E). Kyoto Encyclopedia of Genes and Genomes (KEGG) enrichment analysis was performed using the DEGs upregulated in Control cells. We found that the Wnt signaling pathway was one of the top 5 enriched pathways (Fig. 4F). These DEGs were marked in red color in the Wnt signaling pathway schematic diagram generated by KEGG browser, revealing the expressions of canonical *Wnt* ligands, receptors like *Frizzled*, and direct target like *Axin* were decreased in *Gem*-Kd cells (Additional file 1: Figure S3). These findings suggested that Wnt signaling was suppressed in *Gem*-Kd cells during chondrogenic differentiation.

### Reversion effects Wnt activation during chondrogenesis in *Gem*-KD cells

**Fig. 5:**
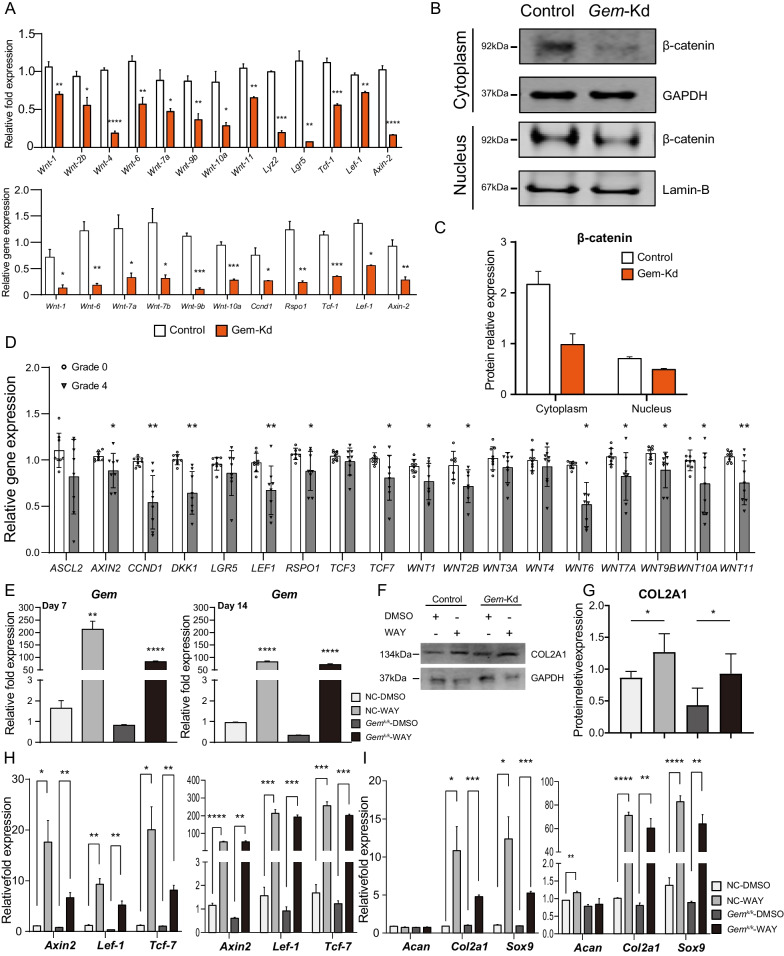
Wnt activation reverses the effects of Gem silence on chondrogenesis. **A** The mRNA expression of multiple genes involved in Wnt signaling in Gem-Kd cells compared to Control cells 3 days and 21 days after chondrogenic induction. **B** Immunoblot of β-CATENIN in nucleus and cytoplasm in the Gem-Kd cells compared to the Control cells. **C** Quantitative analysis of Immunoblot. **D** The mRNA expressions of WNT and relative genes in Grade 0(normal) and Grade 4 (OA) samples. **E** The mRNA expression of Gem among Control and Gem-Kd ATDC5 cells 7 and 14 days after treated with Wnt agonist (WAY). **F** Immunoblot of COL2A1 among Control and Gem-Kd ATDC5 cells 7 days after treated with Wnt agonist (WAY). **G** Quantitative analysis of Immunoblot. **H **The mRNA expressions of Axin2, Lef-1, and Tcf-7 among Control and Gem-Kd ATDC5 cells 7 and 14 days after treated with Wnt agonist (WAY). **I** The expressions of chondrogenic markers Acan, Sox9, and Col2a1 among Control and Gem-Kd ATDC5 cells 7 and 14 days after treated with Wnt agonist (WAY). All experiments were repeated at least for three times. All data are expressed as mean ± SEM. *p < 0.05, **p < 0.01, ***p < 0.001, ****p < 0.001

To verify the RNA sequencing results, real-time PCR was utilized to detect the expressions of multiple associated genes of Wnt signaling in Gem-Kd cells 3 and 21 days after chondrogenic induction (Fig. 5A). We found that the expressions of *Axin2*, *Lef-1*, and *Tcf-7* were decreased in *Gem*-Kd cells compared to controls both 3 and 21 days after induction. Protein of cytoplasm and nucleus extracted from *Gem*-Kd cells showed lower β-CATENIN level (Fig. 5B, C, Additional file 1: Figure S4A–D). These results validated the findings uncovered by RNA sequencing analysis. In addition, we performed qPCR to investigate the WNT and relative genes in Grade 0 (normal) and Grade 4 (OA) samples. We found that the expressions of multiple associated genes of Wnt signaling were decreased in OA cartilage compared to controls (Fig. 5D). Moreover, we tried to stimulate *Gem*-Kd cells with exogenous Wnt ligands (WAY) to reverse the suppression of Wnt signaling. In parallel, Control cells (NC) and Gem-Kd cells were treated with DMSO or WAY. The expressions of *Gem* were upregulated in WAY treated groups 7 and 14 days after induction (Fig. 5E). The expressions of *Axin2*, *Lef-1*, and *Tcf-7* were also significantly increased in WAY treated cells (Fig. 5H), suggesting successful reversion of the activity of Wnt signaling in *Gem*-Kd cells by WAY. The mRNA expressions of chondrogenic markers such as *Col2a1* and *SOX9* were increased in WAY treated cells compared to DMSO treated cells (Fig. 5I). We also found that the protein expression of COL2A1 was increased in WAY treated cells compared to DMSO treated cells (Fig. 5F–G), which was consistent with those obtained by qPCR. Taken together, these findings suggested that loss of function of *Gem* suppressed chondrogenic differentiation and cartilage matrix formation by downregulating Wnt/β-catenin signaling.

## Discussion

OA is a highly prevalent chronic disorder of joints [26] characterized by progressive breakdown of the extracellular matrix and accelerated loss of articular cartilage [27]. A number of factors contribute to osteoarthritis cartilage destruction, including abnormal biomechanics, injuries, overloads, and instability, resulting in an imbalance between anabolic and catabolic factors in cartilage [28, 29]. In previous research, we successfully constructed materials to promote cartilage repair [30]. However, the cartilage tissue induced by this recovery was of different structure compared to the natural cartilage tissue. To date, there is no clinical proof treatment to reverse cartilage loss in OA [31]. The formation of cartilage tissue includes two processes: differentiation of chondrocytes and formation of extracellular matrix by mature chondrocytes [32]. Although multiple genes have been identified as regulators during chondrogenesis, more mechanisms underlying this process in OA conditions still urgent further studies.

Our study found decreased expression of GEM in OA patients’ cartilage tissues and TBHP-treated ATDC5 cells, identifying GEM as a potentially protective factor in OA amelioration. We also treated the C28/I2 cell line and mouse primary chondrocytes with TBHP approaches to mimic OA pathogenic alteration and observed consistent results. Next, we found that GEM silencing suppressed chondrogenic differentiation with downregulated expression of chondrogenesis and cartilage matrix formation markers in ATDC5 cells. Furthermore, we utilized RNA sequencing to identify the potential mechanism underlying the regulatory effect of Gem on chondrogenic differentiation. We found that canonical Wnt signaling might contribute to the detrimental effects of GEM loss on cartilage differentiation. We verified this finding by reversing the suppression of Wnt signaling by exogenous Wnt activation. Therefore, these results collectively validated that GEM functions as a novel regulator mediating chondrogenic differentiation and cartilage matrix formation through the canonical Wnt signaling pathway.

A growing number of studies indicate that GEM participates in multiple biological functions. There are different structural sites in the GEM protein as a member of the RGK family [33]. It has been reported that GEM is involved in rearrangement of the cytoskeleton, which is mediated by ROK7 [34]. Anastassia et al. found that GEM protein interacted with the membrane–cytoskeleton linker protein ezrin in its active state and induced cell elongation [35]. Moreover, we now report that GEM plays a crucial role in regulating the differentiation of chondrocytes and matrix formation. Another member of the RGK family, RAD, has also been shown to be required for normal bone homeostasis in mice, and deletion of RAD in mice results in low bone density [36]. In this study, we found that GEM may be necessary for cartilage development.

A variety of diseases are influenced by Wnt signaling cascades, which modulate biological processes including early embryonic development, organogenesis, growth as well as postnatal tissue homeostasis [37]. Several signaling cascades are activated by Wnt proteins. The best understood pathway is the so-called ‘canonical’ Wnt signaling pathway, resulting in the translocation of β-catenin to the nucleus. Other cascades are collectively classified as ‘noncanonical.’ It is evident that the Wnt/b-catenin pathway displays a great deal of complexity and fine-tuning, and many aspects of its regulation are still unknown [38]. Finely tuned Wnt signaling pathway is required for cartilage and bone homeostasis: in rodent models, both activation and suppression of the Wnt–β-catenin cascade can cause osteoarthritis [39, 40]. It has been reported that Wnts are crucial for articular cartilage and bone homeostasis, as we found a decreased expression of Wnt signaling in human OA cartilage compared to controls. But some other researchers investigated that cartilage can be damaged and the stable articular chondrocyte phenotype lost as a result of excessive Wnt signaling [41]. Interestingly, in this study, we revealed that Wnt signaling could be depressed because of GEM knockdown, inducing a loss in cartilage. In 2009, Yusas et al. found that as a result of tamoxifen-driven activation of β-catenin signaling in cartilage, proteoglycans were initially lost in a mouse model, followed by increased cartilage thickness and cell proliferation [42]. The conditional ablation of β-catenin in chondrocytes results in hypocellularity in articular cartilage, which is consistent with the observation that the activity of β-catenin regulates the proliferation of chondrogenic cells. Recently, Giovanna et al. demonstrated that both WNT-3A and the Wnt inhibitor DKK1 induced dedifferentiation in human articular chondrocytes by simultaneously activating β-catenin-dependent and -independent responses. They proposed a novel model in which a single WNT can simultaneously activate multiple pathways with distinct and independent outcomes and with reciprocal regulation [43]. In addition, Bradley et al. found that Wnt5a and Wnt5b promote early chondrogenesis by activating noncanonical Wnt signaling [44, 45]. Hence, when Wnt/β-catenin is both activated and inhibited, mice appear to develop OA-like disease. According to the present study, we discovered that Wnt activation reverses the effects of Gem silencing on chondrogenic differentiation.

Inevitably, there are some limitations to our research. The results we observed came from in vitro experiments. It is true that in vitro experiments bring stable conditions and reduce interference factors, but at the same time, they inevitably ignore the biological complexity of the in vivo environment. Although we have also observed the expression of GEM from tissues of human patients, we still need to reproduce this process in animal experiments to obtain more comprehensive biological changes and evidence and to have a deeper understanding of the mechanism of GEM on chondrocyte differentiation and cartilage matrix formation in organisms. As a whole, the Gem-Wnt axis plays a crucial role in regulating the differentiation of chondrocytes and matrix formation to maintain cartilage homeostasis, and targeting Gem in chondrocytes might represent an effective strategy to control OA cartilage disruption.

## Conclusion

Our results collectively validated that GEM functions as a novel regulator mediating chondrogenic differentiation and cartilage matrix formation through Wnt/β-catenin signaling.

### Supplementary Information

**Additional file 1: Figure S1**, also see Figure 1. (**A**) A schematic diagram to represent zoning and grading of femoral and tibial articular cartilage tissues from OA patients. (**B**) The stage of OA tissue and schematic description of OARSI and ICRS grading system for OA cartilage. (**C**) Mankin grades of grade 0 and grade 4 cartilage tissues. (**D**, **E**) Quantitation of immunofluorescence staining of GEM and MMP13 in human knee articular cartilage. (**F**) The mRNA expressions of RRAD and REM of grade 0 and grade 4 cartilage tissues. (**G**) Cell viability of C28/I2 cells after treated with TBHP. All data are expressed as mean ± SEM. *p < 0.05, **p < 0.01, ***p < 0.001, ****p < 0.001. **Figure S2.**, also see Figure 3. (**A**) The mRNA expression of Gem of ATDC5 transfected with control siRNA and three different siRNAs targeting Gem. Si_2 was used in the Gem silencing experiments. (**B**) Alcian Blue staining of Control and Gem-Kd cells 7 and 14 days after chondrogenic induction (scler bar = 100 μm).**Figure S3.**, also see Figure 4. KEGG browser result of Wnt signaling pathway and DEGs upregulated in Control cells were colored in red. **Figure S4.**, also see Figure 5. (**A**, **B**, **C**, **D**) Western blotting detected the protein levels of β-catenin in in nucleus and cytoplasm in Gem-Kd cells compared to controls. **Table S1.** Primer sequences for real-time PCR in this study. **Table S2.** RNA contamination detection
