# Supplementary material for: Decreased expression of GEM in osteoarthritis cartilage regulates chondrogenic differentiation via Wnt/β-catenin signaling
Source: J Orthop Surg Res. 2023 Oct 4;18:751. doi: 10.1186/s13018-023-04236-z (PMC10548561; doi:10.1186/s13018-023-04236-z)
Supplement: Supplementary file 1 — Additional file 1: Figure S1, also see Figure 1. (A) A schematic diagram to represent zoning and grading of femoral and tibial articular cartilage tissues from OA patients. (B) The stage of OA tissue and schematic description of OARSI and ICRS grading system for OA cartilage. (C) Mankin grades of grade 0 and grade 4 cartilage tissues. (D, E) Quantitation of immunofluorescence staining of GEM and MMP13 in human knee articular cartilage. (F) The mRNA expressions of RRAD and REM of grade 0 and grade 4 cartilage tissues. (G) Cell viability of C28/I2 cells after treated with TBHP. All data are expressed as mean ± SEM. *p < 0.05, **p < 0.01, ***p < 0.001, ****p < 0.001. Figure S2., also see Figure 3. (A) The mRNA expression of Gem of ATDC5 transfected with control siRNA and three different siRNAs targeting Gem. Si_2 was used in the Gem silencing experiments. (B) Alcian Blue staining of Control and Gem-Kd cells 7 and 14 days after chondrogenic induction (scler bar = 100 μm).Figure S3., also see Figure 4. KEGG browser result of Wnt signaling pathway and DEGs upregulated in Control cells were colored in red. Figure S4., also see Figure 5. (A, B, C, D) Western blotting detected the protein levels of β-catenin in in nucleus and cytoplasm in Gem-Kd cells compared to controls. Table S1. Primer sequences for real-time PCR in this study. Table S2. RNA contamination detection [file 13018_2023_4236_MOESM1_ESM.pdf]

Supplementary Figure 1, also see Figure 1

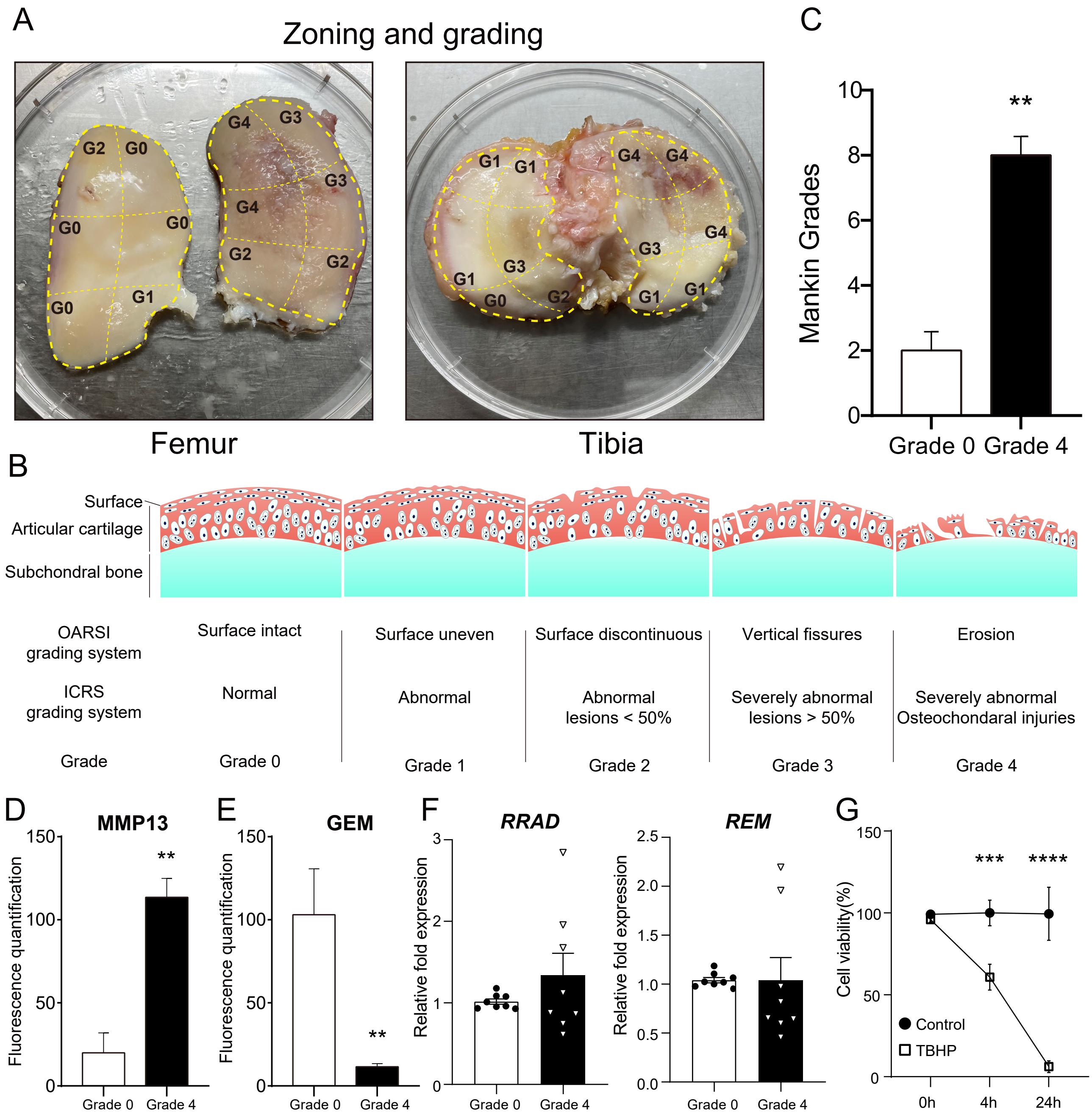

Supplementary Figure 2, also see Figure 3

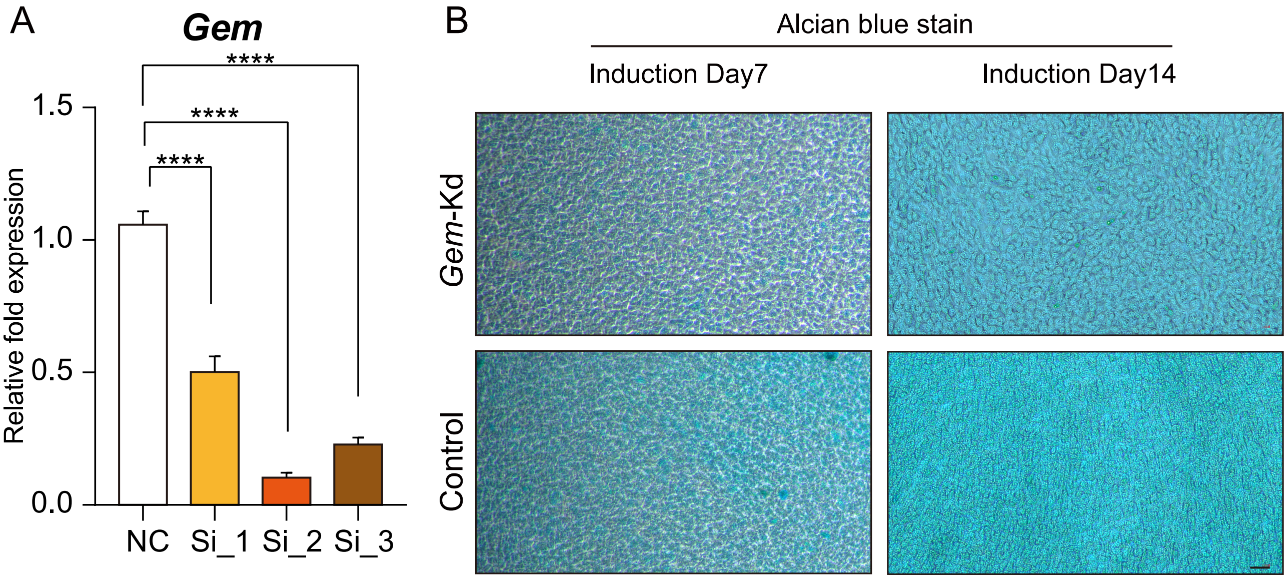

Supplementary Figure 3, also see Figure 4

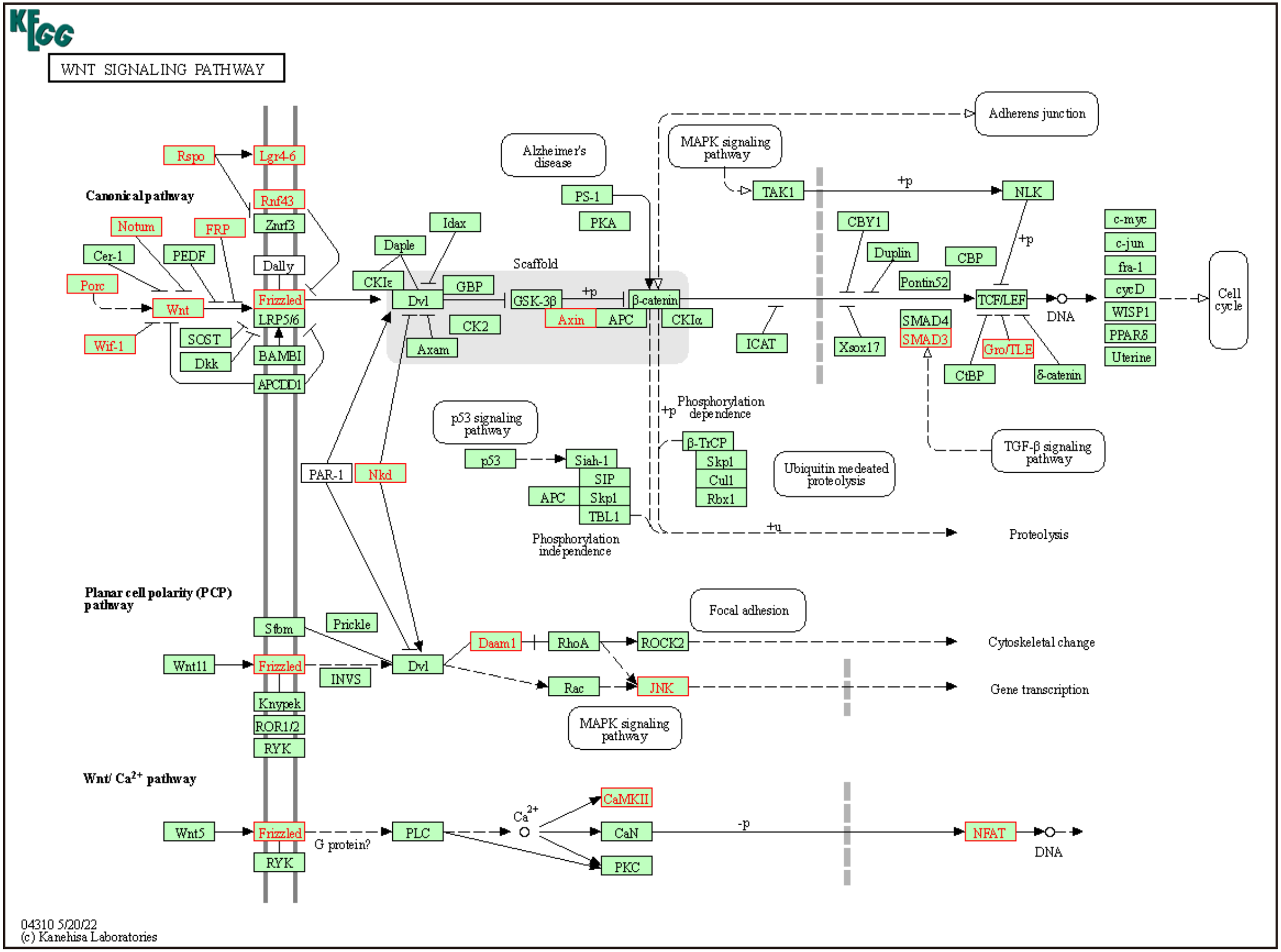

Supplementary Figure 4, also see Figure 5

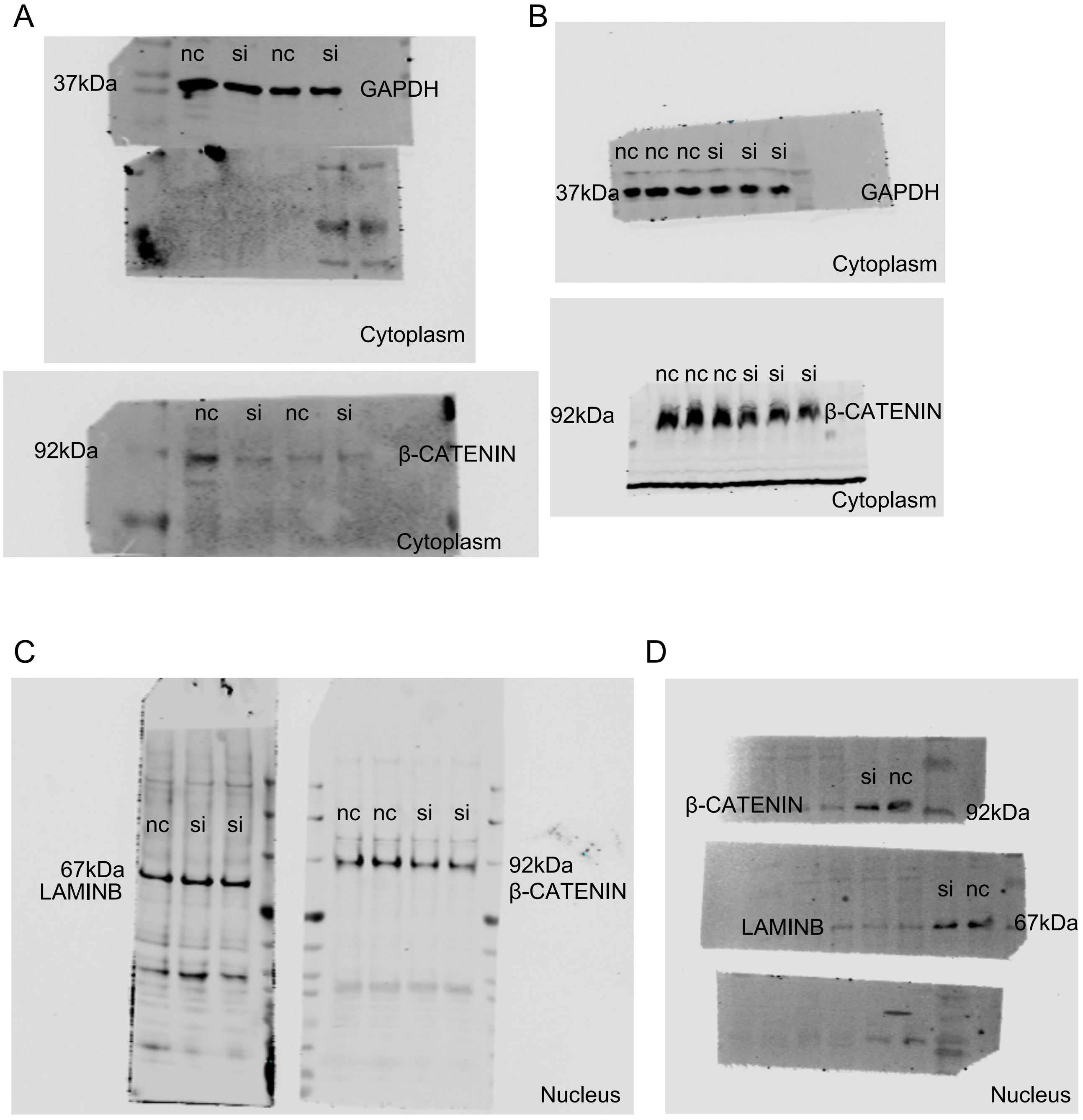

Supplement Table 1

Primer name	Sequence
**hGEM Fw**	CATGACAGCATGGACAGCGA
**hGEM Rv**	GGTTGCACTCTCTCCGTCAA
**hSox9 Fw**	AGCGAACGCACATCAAGAC
**hSox9 Rv**	CTGTAGGCGATCTGTTGGGG
**hCol2a1 Fw**	TGGACGATCAGGCGAAACC
**hCol2a1 Rv**	GCTGCGGATGCTCTCAATCT
**hACAN Fw**	ACTCTGGGTTTTCGTGACTCT
**hACAN Rv**	ACACTCAGCGAGTTGTCATGG
**hREM Fw**	TGCCTTCCACTCAATCCCAG
**hREM Rv**	GATCTCCAAGTAGCACCACAC
**mGem Fw**	CATGACAGCATGGACAGCGA
**mGem Rv**	GGTTGCACTCTCTCCGTCAA
**mSox9 Fw**	ATGAAGATGACCGACGAGCA
**mSox9 Rv**	AACTTGTCCTCCTCGCTCTC
**mCol2a1 Fw**	GATGACTTTCCTCCGTCTACTGTCC
**mCol2a1 Rv**	GTATGTGAACCTGCTGTTGCCC
**mAcan Fw**	GAGACTTCTGCCTCTGGAATAG
**mAcan Rv**	CTCCAGAAGGAATCCCACTAAC
**mAxin2 Fw**	AAGGTCCTGGCAACTCAGTA
**mAxin2 Rv**	ATGTGAGCCTCCTCTCTTTTAC
**mTcf7 Fw**	CCGTATCCACAACAGCTTGC
**mTcf7 Rv**	GGCCGCTGATTCTTTCAAGG
**mLef1 Fw**	CGGGAAGAGCAGGCCAAATA
**mLef1 Rv**	TCTGGGACCTGTACCTGAAGT
**mWnt1_Fw**	GGGTTTCTACTACGTTGCTACT
**mWnt1_Rv**	CAGACTCTTGGAATCCGTCAA

Primer name	Sequence
**mWnt2b_Fw**	GAGTGATCTGTGACAACATCCC
**mWnt2b_Rv**	TGACGAGATAGCATAGACGAAC
**mWnt4_Fw**	AGTTCAAGCCACATACAGATGA
**mWnt4_Rv**	TTTAGATGTCTTGTTGCACGTG
**mWnt6_Fw**	CGGTCACTCAAGCCTGTTCCATG
**mWnt6_Rv**	GAGGTCCAGGAGTGCCCAGAAG
**mWnt7a_Fw**	TGCCTTCACCTATGCGATTATC
**mWnt7a_Rv**	CGCCTCGTTATTGTGTAAGTTC
**mWnt7b_Fw**	CTCAAGGAGAAGTACAACGCAG
**mWnt7b_Rv**	GTCTCCATAGGCTTCTGGTAG
**mWnt9b_Fw**	CTCCTGTGCTGTTCGTACCTGTTG
**mWnt9b_Rv**	GCCGTGTCATAGCGTAGCTTCAG
**mWnt10a_Fw**	AGGTTTTCGAGAGAGTGCTTTC
**mWnt10a_Rv**	AAGCCTTCAGTTTACCCAGAG
**mWnt11_Fw**	AATCAGACGCAACACTGTAAAC
**mWnt11_Rv**	CTCGATGGAGGAGCAGTTC
**mRspo1_Fw**	CATTCTGCTGGAGAGGAACGACATC
**mRspo1_Rv**	GCCTCACAGTGCTCGATCTTGC
**mLgr5_Fw**	TTTTTCTGTCAAGTGCTCTTCG
**mLgr5_Rv**	CAGGACACATAGCAAAACGATC
**mCcnd1_Fw**	CGTATCTTACTTCAAGTGCGTG
**mCcnd1_Rv**	ATGGTCTCCTTCATCTTAGAGG
**mLyz2_Fw**	AGGTCTATGAACGTTGTGAGTT
**mLyz2_Rv**	ACCAGTATCGGCTATTGATCTG

Supplement Table 2

sample	concentration （ng/uL）	A260/A280	A260/A230
C28/I2-0h-1	176.2	1.86	2.34
C28/I2-0h-2	159.9	1.88	2.32
C28/I2-0h-3	168.2	1.78	2.34
C28/I2-TBHP-4h-1	185.5	1.89	2.37
C28/I2-TBHP-4h-2	162.2	1.91	2.06
C28/I2-TBHP-4h-3	171.2	1.95	1.94
C28/I2-TBHP-24h-1	158.4	1.84	2.35
C28/I2-TBHP-24h-2	169.8	1.9	1.98
C28/I2-TBHP-24h-3	160.6	1.9	1.91
ATDC5-0h-1	192.5	2.04	2.07
ATDC5-0h-2	199.7	1.86	2.19
ATDC5-0h-3	189.8	1.91	2.18
ATDC5-TBHP-4h-1	166.5	1.94	2.1
ATDC5-TBHP-4h-2	156.2	1.96	2.26
ATDC5-TBHP-4h-3	149.8	1.9	2.01
ATDC5-TBHP-24h-1	159.6	1.89	2.34
ATDC5-TBHP-24h-2	156.2	1.9	2.32
ATDC5-TBHP-24h-3	156.6	1.98	2.06
Primary-0h-1	129.5	2	2.3
Primary-0h-2	108.6	1.95	2.35
Primary-0h-3	117.8	1.93	2.08
Primary-TBHP-4h-1	118.2	1.91	2.32
Primary-TBHP-4h-2	119.1	1.89	2.32
Primary-TBHP-4h-3	115.6	1.9	2.04
Primary-TBHP-24h-1	128.3	1.91	2.18
Primary-TBHP-24h-2	134.6	1.95	2.1
Primary-TBHP-24h-3	119.3	1.84	2.25
